# Structural–Functional Customization of Nanoscale Liposome-in-Liposome Systems: Precision Engineering Methodology and Artificial-Intelligence-Driven Design Prospects

**DOI:** 10.34133/research.1168

**Published:** 2026-02-19

**Authors:** Liutao Hu, Jianfeng Cai, Chao Lu

**Affiliations:** ^1^ State Key Laboratory of Bioactive Molecules and Druggability Assessment, Guangdong Basic Research Center of Excellence for Natural Bioactive Molecules and Discovery of Innovative Drugs, College of Pharmacy, Jinan University, Guangzhou 511436, China.; ^2^ Department of Chemistry, University of South Florida, Tampa, FL 33620, USA.

## Abstract

Liposome-in-liposome systems with multicompartment structures offer advantages in structural complexity and functional programmability, but their application is limited by poor controllability in conventional fabrication. A recent Nature Chemistry study by Elani et al. reports a methodology to prepare sub-200-nm dual-layered liposomes, enabling customizable bilayers and adjustable interbilayer spaces. Their stepwise assembly approach promises to facilitate the integration of artificial intelligence expertise from unilamellar liposome research into liposome-in-liposome systems, thereby further optimizing formulations and enabling biological predictions. Consequently, this advancement may accelerate the clinical translation of liposomal drugs and advance frontier research in areas such as artificial organelles and lipid nanoparticles.

Liposomes are prominent nanodrug delivery systems due to their commercial success and versatility, with potential applications as chemical reactors, synthetic organelles, biosensors, and biological membrane models [1–3]. Their phospholipid bilayer mimics cell membranes, enabling efficient encapsulation of hydrophilic and hydrophobic drugs, high biocompatibility, and easy surface modification for targeted delivery. As clinical requirements grow complex, multicompartment liposome-in-liposome systems show superior potential over monolayer vesicles for combination therapies and controlled release. These systems include multilamellar vesicles (MLVs) with concentric bilayers and multivesicular liposomes (MVLs) containing multiple internal vesicles. Traditionally, MLVs are fabricated via thin-film hydration and MVLs through a double emulsion method [1]. These processes rely on spontaneous assembly and drug loading through phospholipid–drug interactions, resulting in unpredictable layer numbers and composition, broad size distribution, low encapsulation efficiency, and limited reproducibility. Consequently, conventional fabrication restricts liposome architectural complexity and functional versatility.

Recently, Elani and colleagues [4] from Imperial College London reported in Nature Chemistry a microfluidic strategy using click chemistry to produce nanosized MLVs termed concentrisomes (≈120 nm; polydispersity index ≈ 0.26) (Fig. 1). These MLVs contain 2 distinct compartments, enabling multistage release and in situ biochemical synthesis. Specifically, alkyne-functionalized preformed liposomes flow into a microfluidic hydrodynamic focusing chip alongside an ethanol solution of azide-functionalized lipids. Micellar or bicellar aggregates attach to the external leaflet of the preformed liposomes and then close to form a second bilayer. Click chemistry adjusts the interbilayer space using azide- and alkyne-functionalized polyethylene glycol (PEG) scaffolds, crucially increasing the double-bilayer ratio to 60%. Cryogenic transmission electron microscopy (cryo-TEM) and dynamic light scattering confirmed the formation of the second bilayer, highlighting click chemistry’s role in modulating concentrisome structure and preparation efficiency.

**Fig. 1.**
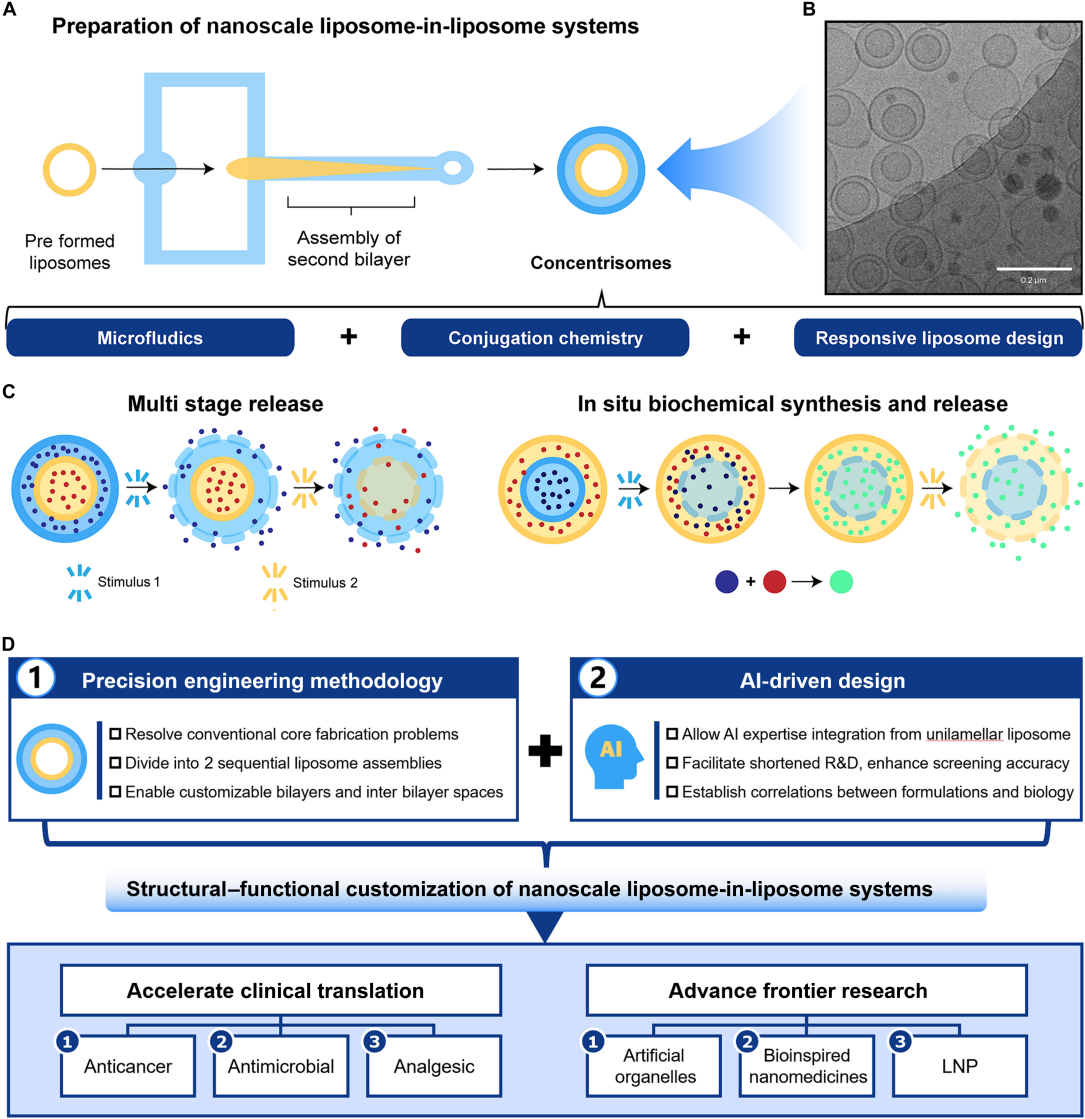
Nanoscale liposomes-in-liposomes systems that support multistage release and in situ biochemical synthesis. (A) Schematic diagram of the preparation method of nanosized concentrisomes by combining microfluidics and click chemistry. (B) Representative cryo-TEM micrograph of concentrisomes. Reprinted with permission from [4]. Copyright 2025, Springer. (C) The graphical representation shows the concentrisome’s capability to encapsulate multiple cargos in separate compartments, followed by the sequential multistage release of 2 different payloads, and to perform in situ biochemical synthesis within its attoliter volume, followed by release. (D) Schematic illustration of nanoscale liposome-in-liposome systems developed by combining precision engineering methodology with AI-driven design to accelerate clinical translation and advance frontier research.

This study investigated multistage release in concentrisomes by regulating bilayer composition and drug loading, demonstrating that temperature changes control sequential release from compartments. For example, concentrisomes with bilayers sensitive to 52 and 42 °C encapsulated methylene blue in the outer compartment and calcein in the inner, enabling methylene blue release at 42 °C, followed by calcein at 52 °C upon heating. Using analogous principles, concentrisomes were tested as attoliter reactors for biochemical synthesis, with fluorescein di-β-d-galactopyranoside in a thermoresponsive inner compartment and β-galactosidase in a nonresponsive outer compartment. At 42 °C, the inner membrane became permeable, allowing substrate and enzyme interaction to initiate the reaction.

The work of Elani et al. [4] represents a important advancement in nanoscale liposome-in-liposome precision engineering. While microfluidics has been used previously, this approach outperforms conventional techniques that typically yield only unilamellar liposomes. Thin-film hydration yields multicompartment structures but offers poor control, whereas the conventional double-emulsion method for MVLs produces large liposomes via complex steps. Even 3-channel microfluidic devices can produce multicompartment double emulsions, but they are inherently unstable and typically yield giant unilamellar liposomes (>20 μm) rather than nanostructures [5]. Consequently, achieving nanoscale liposome-in-liposome systems with customizable membrane structure and function remains challenging. Elani et al. [4] addressed this using a dual-channel microfluidic device, integrating 2 distinct preparation processes with click chemistry to precisely engineer sub-200-nm dual-layered liposomes with customizable membrane compositions and adjustable interbilayer spaces. This innovation overcomes issues of instability, limited customization, and large size (0.5 to 5 μm for MLVs and 1 to 100 μm for MVLs) in traditional fabrication [1], enabling multifunctional, programmable, and scalable applications.

The customizable nanoscale liposome-in-liposome methodology promises enhanced delivery of anticancer agents, antimicrobials, analgesics, protein/peptide drugs, and nucleic acid therapeutics. It may meet clinical needs including precise combination therapies, minimized side effects, sustained and prolonged release, and personalized delivery. In cancer treatment, MLVs/MVLs allow simultaneous delivery and controlled release of multiple drugs, overcoming efficacy loss and side effects from drug metabolism differences in traditional therapies [6]. Against antibiotic-resistant infections, they allow sequential release of adjuvants and antibiotics (e.g., membrane-disruptive agents before antimicrobials) to better manage hospital-acquired infections [7]. In pain management, liposome-in-liposome systems could offer prolonged, stimulus-responsive drug release at inflammation sites, reducing opioid-related respiratory depression risks and improving safety [8]. The US Food and Drug Administration has approved micrometer-sized products include Exparel, DepoCyte, and DepoDur, with drugs such as dexmedetomidine under investigation using MVL technology. The translational potential of MLVs/MVLs highlights the significance of customizing their structure and function, while nanoscale advancements could overcome microscale limitations such as poor targeting and permeability.

Furthermore, nanoscale liposome-in-liposome technology offers promising advancements in nanomedicine, biology, and materials science by enabling cross-disciplinary applications [9,10]. Its multichamber designs could create programmable artificial organelles mimicking natural functions for enzyme cascades, energy generation, and metabolic regulation [11]. In addition, its dual-layer design supports bioinspired structures, such as viral-mimetic architectures, with an outer layer for environmental protection and an inner layer for targeted drug release, emulating natural defenses [12]. For mRNA vaccine challenges such as degradation susceptibility and excessive immunogenicity, the dual-layer lipid nanoparticle (LNP) design reduces immunogenicity via outer PEG modification while enabling pH-triggered mRNA release in endosomes. It balances delivery efficiency and safety through layered lipid adjustments and supports mRNA-adjuvant codelivery via multicompartmental structures [13].

To advance nanoscale liposome-in-liposome systems toward clinical applications, artificial intelligence (AI) and machine learning (ML) are essential for optimization. ML tackles challenges including labor-intensive nanoparticle synthesis and limited nano–bio interaction understanding [14], while enhancing detection, imaging, and therapeutic applications. Such AI-driven approaches complement emerging techniques such as bridging nanocarrier design and clinical translation of Elani et al. [4] They surpass traditional computational methods by intelligently integrating multisource data, adaptively optimizing carrier design and delivery pathways, and precisely predicting release kinetics and targeting performance. This synergy enhances liposomal research and development (R&D) efficiency, reduces costs, and enables personalized therapies [15].

Specifically, AI shortens liposome R&D cycles and costs, while improving formulation screening accuracy. ML develops predictive models for liposome formulations, forecasting parameters such as size, polydispersity, zeta potential, and encapsulation efficiency [16]. AI modeling cuts down experimental iterations and material waste by optimizing resource use. It also predicts lipid behavior in microfluidic production, despite inherent challenges related to formulation-specific responses to process conditions and chip design variability. ML-based models, notably the XGBoost algorithm, can predict critical quality attributes and process parameters for microfluidics-generated liposomes [17]. Extensive experiments validate the reliability and generalizability of such approaches for determining key microfluidic parameters such as flow rate ratios and resultant liposome size.

Beyond production, AI links nanoparticle formulations to biological behaviors such as nanotoxicity, pharmacokinetics, and fate. For example, ML models accurately predict inorganic nanomaterial cytotoxicity across diverse cell lines [18]. Integrating AI-driven quantitative structure–activity relationship models with physiologically based pharmacokinetic models enables precise nanoparticle pharmacokinetic profile prediction in tumors post-intravenous-administration [19]. Furthermore, neural networks trained on protein corona proteomic data can predict nanoparticle blood clearance and organ accumulation, demonstrating AI’s ability to elucidate complex interactions between nanoparticle surface properties and biological outcomes [20].

Overall, liposome-in-liposome methodology of Elani et al. [4] uses separate steps for preformed liposomes and second bilayer assembly, enabling rapid integration of AI expertise from unilamellar liposome development into R&D. Consequently, nanoscale liposome-in-liposome systems may gain markedly from merging advanced preparation technologies with AI-driven design, shortening R&D time and improving precision, reproducibility, and scalability. With further clinical validation, these systems could become key in next-generation drug delivery, advancing nanotheranostics and transforming personalized medicine, biosynthesis, and diagnostics.

Nevertheless, microfluidic preparation heavily depends on click chemistry and modified lipids, potentially raising costs and slowing scale-up. AI-driven design suffers from scarce dynamic biological datasets, risking discrepancies between predicted and actual biological effects in complex physiological settings. Clinical translation demands enhanced system stability, better comprehension of dynamic in vivo behavior, and reduced immunogenicity to ensure efficacy and safety. Addressing these challenges requires further innovation.
